# Study Protocol of the Korean EGFR Registry: A Multicenter Prospective and Retrospective Cohort Study in Nonsmall Cell Lung Cancer Patients With EGFR Mutation

**DOI:** 10.1111/crj.70043

**Published:** 2025-01-05

**Authors:** Chang Dong Yeo, Dong Won Park, Seong Hoon Yoon, Eun Young Kim, Jeong Eun Lee, Shin Yup Lee, Chang‐Min Choi, In‐Jae Oh, Do Jin Kim, Jeong Seon Ryu, Jae Cheol Lee, Young‐Chul Kim, Tae Won Jang, Kye Young Lee, Seung Hun Jang, Seung Joon Kim, for the Korean EGFR Registry Investigators, Chi Young Kim, In Ae Kim, Ji Woong Son, Seung Hyeun Lee, Sun Hyo Park, Sui In Choi, Sung Yong Lee, Tae Won Jang, Chi‐Young Jung, Mi‐Hyun Kim, Hyun‐Kyung Lee, Hye Seon Kang, Hyun Jyu Cho, Seung Joon Kim, Jae Cheol Lee, Eun Young Kim, Do‐Jin Kim, So‐My Koo, Seong Hoon Yoon, Chan Kwon Park, June Hong Ahn, Se Hyun Kwak, Sei Hoon Yang, Sang Haak Lee, Jin Woo Kim, Ju Sang Kim, Jun Hyeok Lim, Song Wook Shin, Tae Hoon Kim, Da Hyun Kang, Shin Yup Lee, Ji Young Park, Chang Youl Lee, Dong Won Park, Jin Han Park, In‐Jae Oh, Suk Joong Yong, Sangbong Choi, Sang‐Won Um

**Affiliations:** ^1^ Division of Pulmonary, Critical Care and Sleep Medicine, Department of Internal Medicine, Eunpyeong St. Mary's Hospital, College of Medicine Catholic University of Korea Seoul Republic of Korea; ^2^ Division of Pulmonary Medicine and Allergy, Department of Internal Medicine, Hanyang Medical Center Hanyang University College of Medicine Seoul Republic of Korea; ^3^ Department of Internal Medicine Pusan National University Yangsan Hospital Yangsan Republic of Korea; ^4^ Division of Pulmonology, Department of Internal Medicine, Severance Hospital Yonsei University College of Medicine Seoul Republic of Korea; ^5^ Division of Pulmonology, Department of Internal Medicine, College of Medicine Chungnam National University Daejeon Republic of Korea; ^6^ Department of Internal Medicine Kyungpook National University School of Medicine Daegu Republic of Korea; ^7^ Division of Pulmonary and Critical Care Medicine, Asan Medical Center University of Ulsan College of Medicine Seoul Republic of Korea; ^8^ Department of Internal Medicine Chonnam National University Medical School and Hwasun Hospital Hwasun Republic of Korea; ^9^ Division of Respiratory‐Allergy, Department of Internal Medicine, Soonchunhyang University Bucheon Hospital Soonchunhyang University College of Medicine Bucheon Republic of Korea; ^10^ Department of Internal Medicine Inha University Hospital Incheon Republic of Korea; ^11^ Department of Oncology, Asan Medical Center University of Ulsan College of Medicine Seoul Republic of Korea; ^12^ Department of Internal Medicine Kosin University Medical College Pusan Republic of Korea; ^13^ Department of Pulmonary Medicine Konkuk University School of Medicine Seoul Republic of Korea; ^14^ Division of Pulmonary, Allergy and Critical Care Medicine, Department of Internal Medicine, Hallym University Sacred Heart Hospital Hallym University College of Medicine Anyang Republic of Korea; ^15^ Division of Pulmonology, Department of Internal Medicine, Seoul St. Mary's Hospital Catholic University of Korea Seoul Republic of Korea; ^16^ Division of Pulmonology, Department of Internal Medicine Yonsei University College of Medicine Seoul Republic of Korea; ^17^ Department of Internal Medicine Konyang University Hospital Daejeon Republic of Korea; ^18^ Division of Pulmonary, Allergy, and Critical Care Medicine, Department of Internal Medicine, Kyung Hee University Hospital, College of Medicine Kyung Hee University Seoul Republic of Korea; ^19^ Department of Pulmonology, Keimyung University Dongsan Hospital, College of Medicine, Pre‐Medicine The Keimyung University of Korea Daegu Republic of Korea; ^20^ Division of Respiratory and Critical Care Medicine, Department of Internal Medicine Korea University College of Medicine Seoul Republic of Korea; ^21^ Department of Internal Medicine Korea University Medical Center Seoul Republic of Korea; ^22^ Pulmonary Division, Department of Internal Medicine Kosin University Gospel Hospital Busan Republic of Korea; ^23^ Department of Internal Medicine Catholic University of Daegu School of Medicine Daegu Republic of Korea; ^24^ Department of Internal Medicine, Pusan National University Hospital Pusan National University College of Medicine Busan Republic of Korea; ^25^ Division of Pulmonary, Allergy, and Critical Care Medicine, Department of Internal Medicine, Inje University Busan Paik Hospital Inje University College of Medicine Busan Republic of Korea; ^26^ Division of Pulmonary, Allergy and Critical Care Medicine, Department of Internal Medicine, Bucheon St. Mary's Hospital, College of Medicine Catholic University of Korea Seoul Republic of Korea; ^27^ Division of Pulmonary and Critical Care Medicine, Department of Medicine, Samsung Changwon Hospital Sungkyunkwan University School of Medicine Suwon Republic of Korea; ^28^ Division of Pulmonary Medicine, Department of Internal Medicine, Seoul St. Mary's Hospital, College of Medicine Catholic University of Korea Seoul Republic of Korea; ^29^ Department of Oncology, Asan Medical Center, College of Medicine University of Ulsan Seoul Republic of Korea; ^30^ Department of Internal Medicine Yonsei University College of Medicine Seoul Republic of Korea; ^31^ Division of Respiratory‐Allergy, Department of Internal Medicine, Soonchunhayng University Bucheon Hospital Soonchunhynag University College of Medicine Bucheon Republic of Korea; ^32^ Division of Respiratory‐Allergy Medicine, Department of Internal Medicine, Soonchunhyang University Seoul Hospital Soonchunhyang University College of Medicine Seoul Republic of Korea; ^33^ Division of Pulmonary, Department of Internal Medicine Pusan National University Yangsan Hospital Yangsan Republic of Korea; ^34^ Division of Pulmonary, Allergy and Critical Care Medicine, Department of Internal Medicine, Yeouido St. Mary's Hospital, College of Medicine Catholic University of Korea Seoul Republic of Korea; ^35^ Division of Pulmonology and Allergy, Department of Internal Medicine, Yeungnam University Hospital, College of Medicine Yeungnam University Daegu Republic of Korea; ^36^ Division of Pulmonology, Allergy and Critical Care Medicine, Department of Internal Medicine, Yongin Severance Hospital Yonsei University College of Medicine Seoul Republic of Korea; ^37^ Division of Pulmonary and Critical Care Medicine, Department of Internal Medicine, College of Medicine Wonkwangc University of Korea Seoul Republic of Korea; ^38^ Division of Pulmonary, Critical Care and Sleep Medicine, Department of Internal Medicine, Uijeongbu St. Mary's Hospital, College of Medicine Catholic University of Korea Seoul Republic of Korea; ^39^ Division of Pulmonary and Critical Care Medicine, Department of Internal Medicine, Incheon St. Mary's Hospital, College of Medicine Catholic University of Korea Incheon Republic of Korea; ^40^ Division of Pulmonology, Department of Internal Medicine, Inha University Hospital Inha University College of Medicine Incheon Republic of Korea; ^41^ Division of Pulmonary Medicine, Department of Internal Medicine, College of Medicine Chung Ang University Seoul Republic of Korea; ^42^ Department of Internal Medicine Gyeongsang National University School of Medicine and Gyeongsang National University Changwon Hospital Changwon Republic of Korea; ^43^ Department of Internal Medicine, College of Medicine Chungnam National University Daejeon Republic of Korea; ^44^ Division of Pulmonary and Critical Care Medicine, Department of Internal Medicine, School of Medicine Kyungpook National University Chilgok Hospital Daegu Republic of Korea; ^45^ Division of Pulmonary, Allergy and Critical Medicine, Department of Internal Medicine Hallym University Sacred Heart Hospital Anyang South Korea; ^46^ Division of Pulmonary, Allergy and Critical Care Medicine, Department of Internal Medicine, Chuncheon Sacred Heart Hospital Hallym University Medical Center Chuncheon Republic of Korea; ^47^ Division of Pulmonology and Critical Care Medicine, Department of Internal Medicine, Haeundae Paik Hospital Inje University College of Medicine Busan Republic of Korea; ^48^ Division of Pulmonary Medicine Yonsei University Wonju Severance Christian Hospital Wonju Republic of Korea; ^49^ Division of Pulmonology & Critical Care Medicine, Department of Internal Medicine Inje University Sanggye Paik Hospital Seoul Republic of Korea; ^50^ Division of Pulmonary and Critical Care Medicine, Samsung Medical Center Sungkyunkwan University School of Medicine Seoul Republic of Korea

**Keywords:** EGFR, Korean, lung cancer, registry study

## Abstract

**Introduction:**

The provision of treatment for epidermal growth factor receptor (EGFR)‐mutated nonsmall cell lung cancer (NSCLC) patients has increased in Korea. However, multicenter studies on the clinicopathologic dataset and treatment outcomes, using a large‐scale dataset, have not been conducted. The current study is a prospective and retrospective multicenter observational cohort study that registers all stages of EGFR‐mutated NSCLC patients.

**Methods:**

The Korean EGFR Registry was designed to enroll 2000 patients with all stages of EGFR‐mutated NSCLC from 40 university hospitals across Korea. This study, encompassing both retrospective and prospective cohorts, aims to analyze clinical characteristics, treatment modalities, and outcomes in these patients. Data collection will include patient demographics, smoking history, quality of life assessments, pathological data, and treatment outcomes, with follow‐up until December 2026. The primary endpoint is disease‐free survival in patients who have undergone radical therapy (surgery and radiotherapy) or progression‐free survival in those receiving targeted therapy (first, second, and subsequent lines), chemotherapy (first and subsequent lines), combination therapy, and palliative/maintenance therapy according to stages of EGFR‐mutated NSCLC. The study will explore the diagnostic methods for EGFR mutations, clinical outcomes based on treatment modalities, and metastatic patterns in EGFR‐mutated NSCLC patients. Moreover, it will investigate various aspects, including the safety and efficacy of a new third‐generation EGFR tyrosine kinase inhibitor (TKI), lazertinib, approved for both first‐ and second‐line treatments.

**Discussion:**

This study is expected to provide valuable insights into the epidemiology, risk factors, progression, and treatment outcomes of EGFR‐mutated NSCLC in Korea. The Korean EGFR Registry will contribute significantly to the understanding of the complex dynamics of EGFR‐mutated NSCLC, aiding in the development of more effective and personalized treatment strategies.

## 1 Introduction

Globally, lung cancer is the most prevalent cancer and the leading cause of cancer‐related mortality. Similarly, in Korea, lung cancer remains a leading cause of cancer‐related mortality even though lung cancer treatment has advanced considerably in recent decades [1]. Somatic mutation of the epidermal growth factor receptor (EGFR) gene is a major oncogenic driver in nonsmall cell lung cancer (NSCLC) [2]. Reports suggest that Asians with lung cancer show a higher prevalence of tumor‐associated EGFR mutation [3]. Nationwide data indicate that 36.8% of stage IV Korean lung adenocarcinoma cases in Korea exhibit EGFR mutation, with higher rates among nonsmokers and females [4]. Patients with advanced NSCLC harboring EGFR mutation are treated with EGFR‐tyrosine kinase inhibitors (TKIs), such as gefitinib, erlotinib, afatinib, dacomitinib, and osimertinib, as first‐line treatment leading to high response rates and prolonged progression‐free‐survival (PFS) [5].

There is evidence suggesting that EGFR mutation may lead to a higher risk of metastatic recurrence in patients with adenocarcinoma who have received radical therapy [6]. The phase III ADAURA trial demonstrated that adjuvant osimertinib significantly improved disease‐free survival (DFS) and overall survival (OS) in patients with completely resected, EGFR‐mutated, stage IB to IIIA NSCLC [7]. Several clinicopathologic factors, such as age, sex, smoking history, tumor size, histologic subtype, and the presence of co‐mutations, have been identified as prognosis indicators for recurrence and survival [8]. However, data on early‐stage EGFR‐mutated lung cancer, particularly large‐scale epidemiologic studies, are relatively scares in Korea. Consequently, there is a need for a detailed analysis of clinical outcomes, based on clinicopathologic characteristics and genetic alterations, in patients with resected EGFR‐mutated lung cancer.

Although initial responses to EGFR‐TKIs are dramatic, most of patients eventually developed acquired resistance to EGFR‐TKIs. Furthermore, 20%–30% of patients showed primary resistance despite harboring sensitizing mutation [9]. Approximately half of resistance mechanisms in patients treated with first‐ or second‐generation EGFR TKI are related to the presence of T790M mutation, and the others include MET amplification, ERBB2 amplification, and transformation to small cell lung cancer [10]. Osimertinib, a third‐generation TKI, targets both EGFR sensitizing and T790M mutations. In the FLAURA trial, osimertinib led to superior PFS and longer OS compared to other EGFR‐TKIs (erlotinib or gefitinib) in untreated EGFR‐mutated advanced NSCLC patients [11]. Nonetheless, resistance to osimertinib eventually occurs. C797S and MET alteration are known resistance mechanisms; in the first‐line osimertinib, those patterns are complex and heterogenous [12]. Therefore, predicting resistance development and analyzing resistant patterns and mechanism through a prospective cohort study in patients who have been treated with first‐generation, second‐generation, and third‐generation EGFR‐TKI is crucial.

In this study, we incorporated lazertinib, a new third‐generation EGFR‐TKI. Lazertinib is a novel and potent third‐generation EGFR‐TKI that effectively blocks both EGFR sensitizing and T790M mutations. It has shown significant efficacy and safety in advanced EGFR T790M‐positive NSCLC patients, progressing after first‐ or second‐generation EGFR‐TKIs treatment [13]. The phase III LASER301 trial demonstrated lazertinib to have a significantly higher efficacy compared to gefitinib in the first‐line setting of EGFR‐mutated advanced NSCLC, with a manageable safety profile [14]. Based on the results of these studies, in Korea, lazertinib received approval as a second‐line treatment in July 2021 and subsequently as a first‐line treatment in January 2024. Moreover, lazertinib has also shown excellent blood–brain barrier (BBB) permeability and intracranial efficacy in EGFR‐mutated brain metastasis model [15], indicating its potential effectiveness for EGFR‐mutated NSCLC patients with brain metastasis. This cohort study is expected to significantly contribute to the validation of the effectiveness and safety of lazertinib in both first‐ and second‐line treatment in the real‐world setting.

Brain and bone metastasis are common in EGFR‐mutated NSCLC and often lead to reduced quality of life and poor overall prognosis [16, 17]. Third‐generation EGFR‐TKIs such as osimertinib and lazertinib showed clinically relevant BBB penetration and excellent intracranial efficacy in brain metastasis in EGFR‐mutated NSCLC [18]. However, for patients with asymptomatic brain metastases, determining which patient population with EGFR mutations benefit from local therapy or are better suited for upfront chemotherapy has not yet been clearly defined [19]. Moreover, high prevalence and early occurrence of skeletal‐related events are reported in EGFR‐mutated NSCLC patients before and during TKI treatment, only a small number of retrospective studies have been reported [20, 21].

Although treatment for EGFR‐mutated NSCLC patients is actively being conducted in Korea, there is a lack of large‐scale, multicenter studies focusing on clinicopathologic datasets and real‐world treatment outcomes. Meanwhile, the development of diagnostic tests using various samples is progressing for safe and easy testing, yet their efficacy and utility are still not fully established. Additionally, there exist various unmet needs concerning recurrence and prognosis in patients with EGFR‐mutated NSCLC. Thus, the aim of this study is to collect and analyze the clinicopathologic features, diagnosis, and treatment outcomes of patients by constructing a multicenter registry of patients with EGFR‐mutated NSCLC.

## 2 Material and Methods

### 2.1 Study Design

**FIGURE 1:**
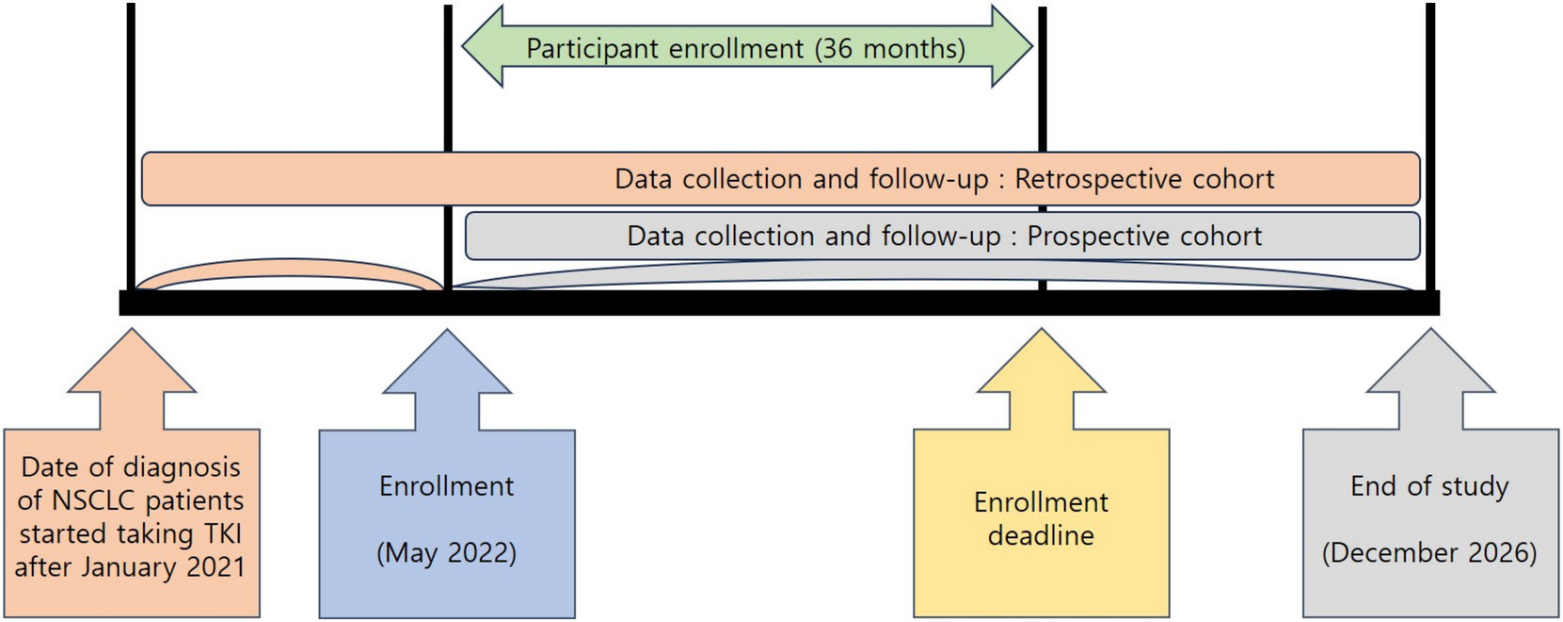
Flow chart of study procedure.

The Korean EGFR Registry is a prospective multicenter observational cohort study including patients with all stages of EGFR‐mutated NSCLC. To address the lengthy follow‐up period required for confirming clinical outcomes, it includes a retrospective cohort comprising recurrent or metastatic NSCLC patients who have been on EGFR‐TKI treatment since January 2021 (Figure 1). The recruitment was planned to take 36 months, starting from May 2022, with follow‐up until December 2026. The study aims to enroll 2000 patients, with 40 university hospitals in Korea participating in a competitive enrollment. This protocol was approved by the Institutional Review Board of all the participating institutes. This study will follow the Declaration of Helsinki as a statement of ethical principles for medical research involving human subjects, including the study of identifiable human substances and data.

### 2.2 Eligibility Criteria

To be included in the study, participants must fulfill the following inclusion criteria and provide informed consent. Patients who do not meet the inclusion criteria will be excluded from the study.

Inclusion criteria:

- Age ≥ 20 years.
- Nationality of South Korea.
- Histologically or cytologically confirmed NSCLC.
- Newly diagnosed NSCLC patients with EGFR mutation (prospective cohort) or recurrent or metastatic NSCLC patients taking EGFR‐TKI after January 2021 (retrospective cohort).

### 2.3 Objectives and Outcome

Primary objective and endpoint:

- Analysis of clinical characteristics and clinical outcomes in NSCLC patients with EGFR mutations.
- DFS in patients undergoing radical therapy (surgery and radiotherapy) or PFS in those receiving targeted therapy (first, second, and subsequent lines), chemotherapy (first and subsequent lines), combination therapy, and palliative/maintenance therapy according to the stages of EGFR‐mutated NSCLC.

Secondary objectives and endpoint:

- Assessment of diagnostic test methods for EGFR mutations in NSCLC patients: frequency and proportion of diagnostic test methods according to various clinical samples.
- Exploration of clinical outcomes based on treatment modality in NSCLC patients with EGFR mutations: OS, overall response rate (ORR), duration of response (DoR), disease control rate (DCR), time to treatment failure (TTF), and time to next treatment (TTNF) according to treatment modality in NSCLC patients with EGFR mutations.
- Exploration of clinical characteristics and metastatic patterns in NSCLC patients with EGFR mutations: analysis of metastatic sites and treatment approaches, particularly for brain, and bone metastasis.
- Safety assessment (particularly for lazertinib): adverse event profiles following lazertinib treatment.

### 2.4 Data Collection and Follow‐Up

Data will be collected by retrieving information from the patient's medical electronic records and through questionnaires filled at baseline and prior to each subsequent treatment. All patients will be followed up until December 2026 to collect long‐term outcome data.

- Patient's characteristics: date of birth, sex, age, comorbidities, cancer history, and family history of cancer.
- Smoking history including electronic cigarette usage.
- Questionnaire for nonsmoking female lung cancer by Korean Association for Lung Cancer [22]: exposure to environmental tobacco smoke or occupational hazards, alcohol consumption history, cooking environment, and use of humidifier disinfectants.
- Quality of life (QoL) questionnaire: EQ‐5D‐5L.
- Pulmonary function tests.
- Pathologic data: date of biopsy, biopsy site, histocytological diagnosis, subtype of EGFR mutations, and other molecular profiles.
- Clinical and pathologic staging: based on the AJCC 8th edition.
- Treatment modality (surgery, radiotherapy, and chemotherapy including EGFR‐TKIs) and clinical outcomes: date of treatment initiation and cessation and reasons for treatment failure.
- Adverse events postlazertinib administration according to the Common Terminology Criteria for Adverse Events version 5.0.
- Vital status at study closure: subject status (alive, death, etc.), date of death, date of withdrawal from the study, and date of last visit.

### 2.5 Statistical Analysis and Sample Size Calculation

Survival analysis methods will be employed to investigate the relationship between pathological variables and survival. Time‐to‐event endpoints will be presented using Kaplan–Meier graphs by treatment modalities. The number of events, median values, and 95% confidence intervals for medians will be presented, and proportions of subjects who did not experience events at 12, 18, 24, and 36 months will be summarized. The hazard ratio (HR) and its 95% confidence interval for the treatment groups will be estimated using the Cox proportional hazard model. For categorical variables, descriptive statistics will be utilized with 95% confidence intervals, assuming a normal distribution. Depending on the analysis objectives, associations will be examined using Pearson's Chi‐square test or Fisher's exact test, and the odds ratio will be estimated using logistic regression analysis. For continuous variables, descriptive statistics will be employed for summarization. If necessary, information related to changes from the baseline, including both magnitude and rate of change, will be presented using descriptive statistics. For safety assessment, adverse events (AE) and serious adverse events (SAE) occurring after the administration of lazertinib will be coded according to the system organ class (SOC) and preferred term (PT) of the Medical Dictionary for Regulatory Activities (MedDRA). The coded data will be presented to demonstrate the number of affected subjects and the incidence rates. A Statistical Analysis Plan (SAP) will provide full details of the statistical analyses.

A sample size of 2000 patients was chosen to ensure a sufficient number for meaningful analysis of various subgroups based on specific stages and treatment modalities. The target sample size is expected to be reasonably achieved through a 3‐year recruitment period across a total of 40 institutions reflecting regional diversity.

### 2.6 Trial Status

As of December 2023, a total of 1600 subjects had been registered, and recruitment was still in progress. We are nearing the completion of participants' inclusion and will continue with follow‐ups until December 2026.

## 3 Discussion

The current study is a combined prospective and retrospective multicenter observational cohort study that registers all stages of EGFR‐mutated NSCLC patients. A long‐term follow‐up utilizing a real‐world dataset is essential to evaluate the clinical outcomes associated with various treatment modalities. To our knowledge, this is the first cohort study in Korea focusing on EGFR‐mutated NSCLC and exploring a range of variables. We also aim to investigate the epidemiology, risk factors, cancer progression, and clinical outcomes according to standard treatment or newly introduced treatment of EGFR‐mutated NSCLC. We believe that the Korean EGFR registry will provide substantial fundamental data and guide future strategies for managing EGFR‐mutated NSCLC patients.

## Author Contributions

All authors had full access to the protocol of the study and take responsibility for the integrity of the protocol. *Conceptualization*: Chang Dong Yeo, Dong Won Park, Seung Hun Jang, and Seung Joon Kim. *Investigation*: Chang Dong Yeo, Dong Won Park, Seong Hoon Yoon, and Eun Young Kim. *Methodology*: Chang Dong Yeo, Dong Won Park, Jeong Eun Lee, and Shin Yup Lee. *Supervision*: Seung Hun Jang and Seung Joon Kim. *Writing – original draft preparation*: Chang Dong Yeo, Dong Won Park, Chang‐Min Choi, and In‐Jae Oh. *Writing – review and editing*: Do Jin Kim, Jeong Seon Ryu, Jae Cheol Lee, Young‐Chul Kim, Tae Won Jang, and Kye Young Lee.

## Conflicts of Interest

The authors declare no conflicts of interest.

## Data Availability

The data that support the findings of this study are available from the corresponding author upon reasonable request.

## Appendix A List of the Korean EGFR Registry Investigators

Chi Young Kim, Division of Pulmonology, Department of Internal Medicine, Yonsei University College of Medicine, Seoul, Republic of Korea.

In Ae Kim, Department of Pulmonary Medicine, Konkuk University School of Medicine, Seoul, Republic of Korea.

Ji Woong Son, Department of Internal Medicine, Konyang University Hospital, Daejeon, 35365, Republic of Korea.

Seung Hyeun Lee, Division of Pulmonary, Allergy, and Critical Care Medicine, Department of Internal Medicine, Kyung Hee University Hospital, College of Medicine, Kyung Hee University, Seoul, Republic of Korea.

Sun Hyo Park, Department of Pulmonology, Keimyung University Dongsan Hospital, College of Medicine, Pre‐Medicine, The Keimyung University of Korea, Daegu, Republic of Korea.

Sui In Choi, Division of Respiratory and Critical Care Medicine, Department of Internal Medicine, Korea University College of Medicine, Seoul, Republic of Korea.

Sung Yong Lee, Department of Internal Medicine, Korea University Medical Center, Seoul, Republic of Korea.

Tae Won Jang, Pulmonary Division, Department of Internal Medicine, Kosin University Gospel Hospital, Busan, Republic of Korea.

Chi‐Young Jung, Department of Internal Medicine, Catholic University of Daegu School of Medicine, Daegu, Republic of Korea.

Mi‐Hyun Kim, Department of Internal Medicine, Pusan National University Hospital, Pusan National University College of Medicine, Busan, Republic of Korea.

Hyun‐Kyung Lee, Division of Pulmonary, Allergy, and Critical Care Medicine, Department of Internal Medicine, Inje University Busan Paik Hospital, Inje University College of Medicine, Busan, Republic of Korea.

Hye Seon Kang, Division of Pulmonary, Allergy and Critical Care Medicine, Department of Internal Medicine, Bucheon St. Mary's Hospital, College of Medicine, The Catholic University of Korea, Seoul, Republic of Korea.

Hyun Jyu Cho, Division of Pulmonary and Critical Care Medicine, Department of Medicine, Samsung Changwon Hospital, Sungkyunkwan University School of Medicine, Republic of Korea.

Seung Joon Kim, Division of Pulmonary Medicine, Department of Internal Medicine, Seoul St. Mary's Hospital, College of Medicine, The Catholic University of Korea, 222, Banpo‐daero, Seocho‐gu, Seoul 06591, Republic of Korea.

Jae Cheol Lee, Department of Oncology, Asan Medical Center, College of Medicine, University of Ulsan, Seoul, Republic of Korea.

Eun Young Kim, Department of Internal Medicine, Yonsei University College of Medicine, Seoul, Republic of Korea.

Do‐Jin Kim, Division of Respiratory‐Allergy, Department of Internal Medicine, Soonchunhayng University Bucheon Hospital, Soonchunhynag University College of Medicine, Bucheon, Republic of Korea.

So‐My Koo, Division of Respiratory‐Allergy Medicine, Department of Internal Medicine, Soonchunhyang University Seoul Hospital, Soonchunhyang University College of Medicine, Seoul, Republic of Korea.

Seong Hoon Yoon, Division of Pulmonary, Department of Internal Medicine, Pusan National University Yangsan Hospital, Yangsan, Republic of Korea.

Chan Kwon Park, Division of Pulmonary, Allergy and Critical Care Medicine, Department of Internal Medicine, Yeouido St. Mary's Hospital, College of Medicine, The Catholic University of Korea, Seoul, Republic of Korea.

June Hong Ahn, Division of Pulmonology and Allergy, Department of Internal Medicine, Yeungnam University Hospital, College of Medicine, Yeungnam University, Daegu, Republic of Korea.

Se Hyun Kwak, Division of Pulmonology, Allergy and Critical Care Medicine, Department of Internal Medicine, Yongin Severance Hospital, Yonsei University College of Medicine, Republic of Korea.

Sei Hoon Yang, Division of Pulmonary and Critical Care Medicine, Department of Internal Medicine, College of Medicine, Wonkwangc University of Korea, Seoul, Republic of Korea.

Sang Haak Lee, Division of Pulmonary, Critical Care and Sleep Medicine, Department of Internal Medicine, Eunpyeong St. Mary's Hospital, College of Medicine, The Catholic University of Korea, Seoul, Republic of Korea.

Jin Woo Kim, Division of Pulmonary, Critical Care and Sleep Medicine, Department of Internal Medicine, Uijeongbu St. Mary's Hospital, College of Medicine, The Catholic University of Korea, Seoul, Republic of Korea.

Ju Sang Kim, Division of Pulmonary and Critical Care Medicine, Department of Internal Medicine, Incheon St. Mary's Hospital, College of Medicine, The Catholic University of Korea, Incheon, Republic of Korea.

Jun Hyeok Lim, Division of Pulmonology, Department of Internal Medicine, Inha University Hospital, Inha University College of Medicine, Incheon, Republic of Korea.

Song Wook Shin, Division of Pulmonary Medicine, Department of Internal Medicine, College of Medicine, Chung Ang University, Seoul, Republic of Korea.

Tae Hoon Kim, Department of Internal Medicine, Gyeongsang National University School of Medicine and Gyeongsang National University Changwon Hospital, Changwon, Republic of Korea.

Da Hyun Kang, Department of Internal Medicine, College of Medicine, Chungnam National University, Daejeon, Republic of Korea.

Shin Yup Lee, Division of Pulmonary and Critical Care Medicine, Department of Internal Medicine, School of Medicine, Kyungpook National University Chilgok Hospital, Daegu, Republic of Korea.

Ji Young Park, Division of Pulmonary, Allergy and Critical Medicine, Department of Internal Medicine, Hallym University Sacred Heart Hospital, Anyang, South Korea.

Chang Youl Lee, Division of Pulmonary, Allergy and Critical Care Medicine, Department of Internal Medicine, Chuncheon Sacred Heart Hospital, Hallym University Medical Center, Chuncheon, Republic of Korea.

Dong Won Park, Division of Pulmonary Medicine and Allergy, Department of Internal Medicine, Hanyang Medical Center, Hanyang University College of Medicine, Seoul, Republic of Korea.

Jin Han Park, Division of Pulmonology and Critical Care Medicine, Department of Internal Medicine, Haeundae Paik Hospital, Inje University College of Medicine, Busan, Republic of Korea.

In‐Jae Oh, Department of Internal Medicine, Chonnam National University Medical School and Hwasun Hospital, Hwasun, Republic of Korea.

Suk Joong Yong, Professor of Internal Medicine, Division of Pulmonary Medicine, Yonsei University Wonju Severance Christian Hospital, 20 Ilsan‐Ro, Wonju‐Si, Gangwon‐Do, 26426, Republic of Korea.

Sangbong Choi, Division of Pulmonology & Critical Care Medicine, Department of Internal Medicine, Inje University Sanggye Paik Hospital, Seoul, Republic of Korea.

Sang‐Won Um, Division of Pulmonary and Critical Care Medicine, Samsung Medical Center, Sungkyunkwan University School of Medicine, Seoul, 06351, Republic of Korea.

## References

[1] J. G. Lee , H. C. Kim , and C. M. Choi , “Recent Trends of Lung Cancer in Korea,” Tuberculosis Respiratory Disease 84, no. 2 (2021): 89–95. 10.4046/trd.2020.0134 PMC8010413 33587838

[2] W. Pao and N. Girard , “New Driver Mutations in Non‐Small‐Cell Lung cancer,” Lancet Oncology 12, no. 2 (2011): 175–180. 21277552 10.1016/S1470-2045(10)70087-5

[3] B. Melosky , K. Kambartel , M. Hantschel , et al., “Worldwide Prevalence of Epidermal Growth Factor Receptor Mutations in Non‐Small Cell Lung Cancer: A Meta‐Analysis,” Molecular Diagnosis & Therapy 26, no. 1 (2022): 7–18. 34813053 10.1007/s40291-021-00563-1 PMC8766385

[4] C. M. Choi , H. C. Kim , C. Y. Jung , et al., “Report of the Korean Association of Lung Cancer Registry (KALC‐R), 2014,” Cancer Research and Treatment 51, no. 4 (2019): 1400–1410. 30913875 10.4143/crt.2018.704 PMC6790858

[5] J. C. Soria , Y. Ohe , J. Vansteenkiste , et al., “Osimertinib in Untreated EGFR‐Mutated Advanced Non‐Small‐Cell Lung Cancer,” New England Journal of Medicine 378, no. 2 (2018): 113–125. 29151359 10.1056/NEJMoa1713137

[6] C. Galvez , S. Jacob , B. S. Finkelman , et al., “The Role of EGFR Mutations in Predicting Recurrence in Early and Locally Advanced Lung Adenocarcinoma Following Definitive Therapy,” Oncotarget 11, no. 21 (2020): 1953–1960. 32523650 10.18632/oncotarget.27602 PMC7260116

[7] M. Tsuboi , R. S. Herbst , T. John , et al., “Overall Survival With Osimertinib in Resected EGFR‐Mutated NSCLC,” New England Journal of Medicine 389, no. 2 (2023): 137–147. 37272535 10.1056/NEJMoa2304594

[8] M. Ito , Y. Miyata , K. Kushitani , et al., “Increased Risk of Recurrence in Resected EGFR‐Positive pN0M0 Invasive Lung Adenocarcinoma,” Thorac Cancer. 9, no. 12 (2018): 1594–1602. 30298562 10.1111/1759-7714.12866 PMC6275825

[9] J. Wang , B. Wang , H. Chu , and Y. Yao , “Intrinsic Resistance to EGFR Tyrosine Kinase Inhibitors in Advanced Non‐Small‐Cell Lung Cancer With Activating EGFR Mutations,” Oncotargets and Therapy 9 (2016): 3711–3726. 27382309 10.2147/OTT.S106399 PMC4922765

[10] D. Westover , J. Zugazagoitia , B. C. Cho , C. M. Lovly , and L. Paz‐Ares , “Mechanisms of Acquired Resistance to First‐ and Second‐Generation EGFR Tyrosine Kinase Inhibitors,” Annals of Oncology 29 (2018): i10–i19. 29462254 10.1093/annonc/mdx703 PMC6454547

[11] S. S. Ramalingam , J. Vansteenkiste , D. Planchard , et al., “Overall Survival With Osimertinib in Untreated, EGFR‐Mutated Advanced NSCLC,” New England Journal of Medicine 382, no. 1 (2020): 41–50. 31751012 10.1056/NEJMoa1913662

[12] J. Chmielecki , J. E. Gray , Y. Cheng , et al., “Candidate Mechanisms of Acquired Resistance to First‐Line Osimertinib in EGFR‐Mutated Advanced Non‐Small Cell Lung cancer,” Nature Communications 14, no. 1 (2023): 1070. 10.1038/s41467-023-35961-y PMC9971254 36849494

[13] B. C. Cho , J. Y. Han , S. W. Kim , et al., “A Phase 1/2 Study of Lazertinib 240 mg in Patients With Advanced EGFR T790M‐Positive NSCLC After Previous EGFR Tyrosine Kinase Inhibitors,” Journal of Thoracic Oncology 17, no. 4 (2022): 558–567. 34958928 10.1016/j.jtho.2021.11.025

[14] B. C. Cho , M. J. Ahn , J. H. Kang , et al., “Lazertinib Versus Gefitinib as First‐Line Treatment in Patients With EGFR‐Mutated Advanced Non‐Small‐Cell Lung Cancer: Results From LASER301,” Journal of Clinical Oncology 41, no. 26 (2023): 4208–4217. 37379502 10.1200/JCO.23.00515

[15] J. Yun , M. H. Hong , S. Y. Kim , et al., “YH25448, an Irreversible EGFR‐TKI With Potent Intracranial Activity in EGFR Mutant Non‐Small Cell Lung Cancer,” Clinical Cancer Research 25, no. 8 (2019): 2575–2587. 30670498 10.1158/1078-0432.CCR-18-2906

[16] M. Ge , Y. Zhuang , X. Zhou , R. Huang , X. Liang , and Q. Zhan , “High Probability and Frequency of EGFR Mutations in Non‐Small Cell Lung Cancer With Brain Metastases,” Journal of Neuro‐Oncology 135, no. 2 (2017): 413–418. 28780743 10.1007/s11060-017-2590-x

[17] A. Brouns , S. Dursun , G. Bootsma , A. C. Dingemans , and L. Hendriks , “Reporting of Incidence and Outcome of Bone Metastases in Clinical Trials Enrolling Patients With Epidermal Growth Factor Receptor Mutated Lung Adenocarcinoma—A Systematic Review,” Cancers (Basel) 13, no. 13 (2021): 3144. 34201833 10.3390/cancers13133144 PMC8267949

[18] M. S. Ahluwalia , K. Becker , and B. P. Levy , “Epidermal Growth Factor Receptor Tyrosine Kinase Inhibitors for Central Nervous System Metastases From Non‐Small Cell Lung Cancer,” Oncologist 23, no. 10 (2018): 1199–1209. 29650684 10.1634/theoncologist.2017-0572 PMC6263119

[19] M. A. Vogelbaum , P. D. Brown , H. Messersmith , et al., “Treatment for Brain Metastases: ASCO‐SNO‐ASTRO Guideline,” Journal of Clinical Oncology 40, no. 5 (2022): 492–516. 34932393 10.1200/JCO.21.02314

[20] M. Lagana , C. Gurizzan , E. Roca , et al., “High Prevalence and Early Occurrence of Skeletal Complications in EGFR Mutated NSCLC Patients With Bone Metastases,” Frontiers in Oncology 10 (2020): 588862. 33282740 10.3389/fonc.2020.588862 PMC7689017

[21] A. Brouns , A. van Veelen , G. D. M. Veerman , et al., “Incidence of Bone Metastases and Skeletal‐Related Events in Patients With EGFR‐Mutated NSCLC Treated With Osimertinib,” JTO Clinical and Research Reports 4, no. 5 (2023): 100513. 37168878 10.1016/j.jtocrr.2023.100513 PMC10165134

[22] Korean Association for Lung Cancer , “Non‐Smoking Female Lung Cancer. Korean Academy of Medical Science: E‐NEWSLETTER No.100,” (2018).

